# SNPs in miRNAs and Target Sequences: Role in Cancer and Diabetes

**DOI:** 10.3389/fgene.2021.793523

**Published:** 2021-12-01

**Authors:** Yogita Chhichholiya, Aman Kumar Suryan, Prabhat Suman, Anjana Munshi, Sandeep Singh

**Affiliations:** Department of Human Genetics and Molecular Medicine, Central University of Punjab, Bathinda, India

**Keywords:** miRNA, microRNA, target genes, seed sequences, SNPs, cancer, diabetes mellitus

## Abstract

miRNAs are fascinating molecular players for gene regulation as individual miRNA can control multiple targets and a single target can be regulated by multiple miRNAs. Loss of miRNA regulated gene expression is often reported to be implicated in various human diseases like diabetes and cancer. Recently, geneticists across the world started reporting single nucleotide polymorphism (SNPs) in seed sequences of miRNAs. Similarly, SNPs are also reported in various target sequences of these miRNAs. Both the scenarios lead to dysregulated gene expression which may result in the progression of diseases. In the present paper, we explore SNPs in various miRNAs and their target sequences reported in various human cancers as well as diabetes. Similarly, we also present evidence of these mutations in various other human diseases.

## Introduction

MicroRNAs (miRNAs) are endogenous single stranded, non-coding, 20–22 nucleotides long molecules that are processed from pre-miRNA. miRNAs have been demonstrated to be tremendously versatile in their function. miRNAs have significant roles in the nucleus as well as cytoplasm in terms of controlling gene expression. They play a significant role in post-transcriptional regulation of gene expression either via translational repression or mRNA degradation (Iorio and Croce, 2012; Peng and Croce, 2016). miRNAs recognize targets by specific base-pairing complementarity between their seed sequence of miRNA (5′ end) and untranslated region (3′UTR) of target gene/mRNA (Ling et al., 2011; Si et al., 2019).

However, in some exceptional cases, base pairing is also reported between 5′ UTR region of the specific mRNA and coding regions (O'Brien et al., 2018; Valinezhad Orang et al., 2014). The standard size of 3′UTR in the human gene is about 950 nucleotides whereas the seed sequence of miRNA is around 6 to 8 nucleotides. The 3′UTR region of a particular mRNA may be recognized by a specific miRNA or by multiple miRNAs. Sequence complementarity is shared by miRNAs with respect to their mRNA targets, resulting in the interaction of a single miRNA with many genes whereas a single gene can probably be regulated by multiple miRNAs (Hashimoto et al., 2013; Mariella et al., 2019).

Around 10 million SNPs are known to be present in both coding as well as non-coding regions of the human genome at a frequency of one in every 300 bp (Moszyńska et al., 2017). Since SNPs have also been reported to be present in seed sequence, it is most likely that the presence of these alterations might disrupt or create new interaction of miRNA with its target site (Palmero et al., 2011; Bhattacharya and Cui, 2017). Furthermore, the SNPs in the 3′UTRs of gene/mRNA can also modulate miRNA-mRNA interactions, protein-mRNA interactions, polyadenylation, all of which might have a serious impact on translation efficiency and mRNA stability (Malhotra et al., 2019a). This in turn might result in the development of various diseases including neurodevelopment disorders, cardiovascular diseases, cancer, autoimmune diseases, and many more (Bruno et al., 2012; Moszyńska et al., 2017).

Cancer and diabetes are multifactorial life threatening human diseases in which various miRNAs have been reported in the pathogenesis as well as the severity of these diseases (Ayaz Durrani et al., 2021). Tens of millions of people are diagnosed with cancer each year around the world, with more than half of those diagnosed dying from it. miRNA profiling and high throughput sequencing in the recent past revealed that miRNA expression is dysregulated in cancer and that its fingerprints might be utilized to classify, diagnose, and prognosis of tumors. miRNAs have been reported to act as oncogenes or tumor suppressors under certain biological conditions. Cancer hallmarks such as sustaining proliferative signals, evading growth suppressors, resisting cell death, activating invasion and metastasis, and initiating angiogenesis have been linked to dysregulated miRNAs (Peng and Croce, 2016).

Diabetes mellitus (DM) affects 347 million people worldwide. Diabetes-related fatalities are expected to double between 2005 and 2030, according to the World Health Organization (Chen et al., 2014a). High blood glucose levels are a defining feature of DM. Diabetes is classified into two types. A deficiency of insulin synthesis in pancreatic cells causes type 1 diabetes (T1D), whereas type 2 diabetes (T2D) is caused by insulin resistance, which causes the body to utilize insulin inefficiently. In both T1D and T2D, long-term hyperglycemia can cause macrovascular (coronary artery disease, peripheral arterial disease, and stroke) as well as microvascular complications (diabetic nephropathy, neuropathy, and retinopathy) (Fowler, 2008). miRNAs are implicated in the etiology and pathogenesis of diabetes and associated complications (Chen et al., 2014a). However, the role of miRNAs in diabetes and its complications are comparatively explored less.

Very few reports are available on SNPs reported in the seed sequence of miRNAs and the 3′UTR region of their target genes. The current review has been compiled with an aim to evaluate the role of genetic variation in the seed sequence of the miRNA and the 3′UTR of their specific target genes in association with the development of the two most common prevalent diseases—cancer and diabetes.

## SNPs in the Seed Sequence of miRNA and the 3′UTR of Specific Target Gene in Cancer

Many miRNAs have been discovered to play a role in the genesis of cancer, either as tumor suppressor genes or as oncogenes. The study of tumor-specific miRNA expression profiles in a variety of malignancies revealed extensive dysregulation of these molecules, some of the overexpression and underexpression of various miRNA (Song and Chen, 2011). As evidenced by multiple findings demonstrating the importance of miRNAs in carcinogenesis, miRNA dysregulation leads to modulation of tumor cell signaling, changes in DNA repair or stress response, and function of the effector protein (Moszyńska et al., 2017; Malhotra et al., 2019a; Galka-Marciniak et al., 2019).

### SNPs in the Seed Sequence of miRNA

**FIGURE 1:**
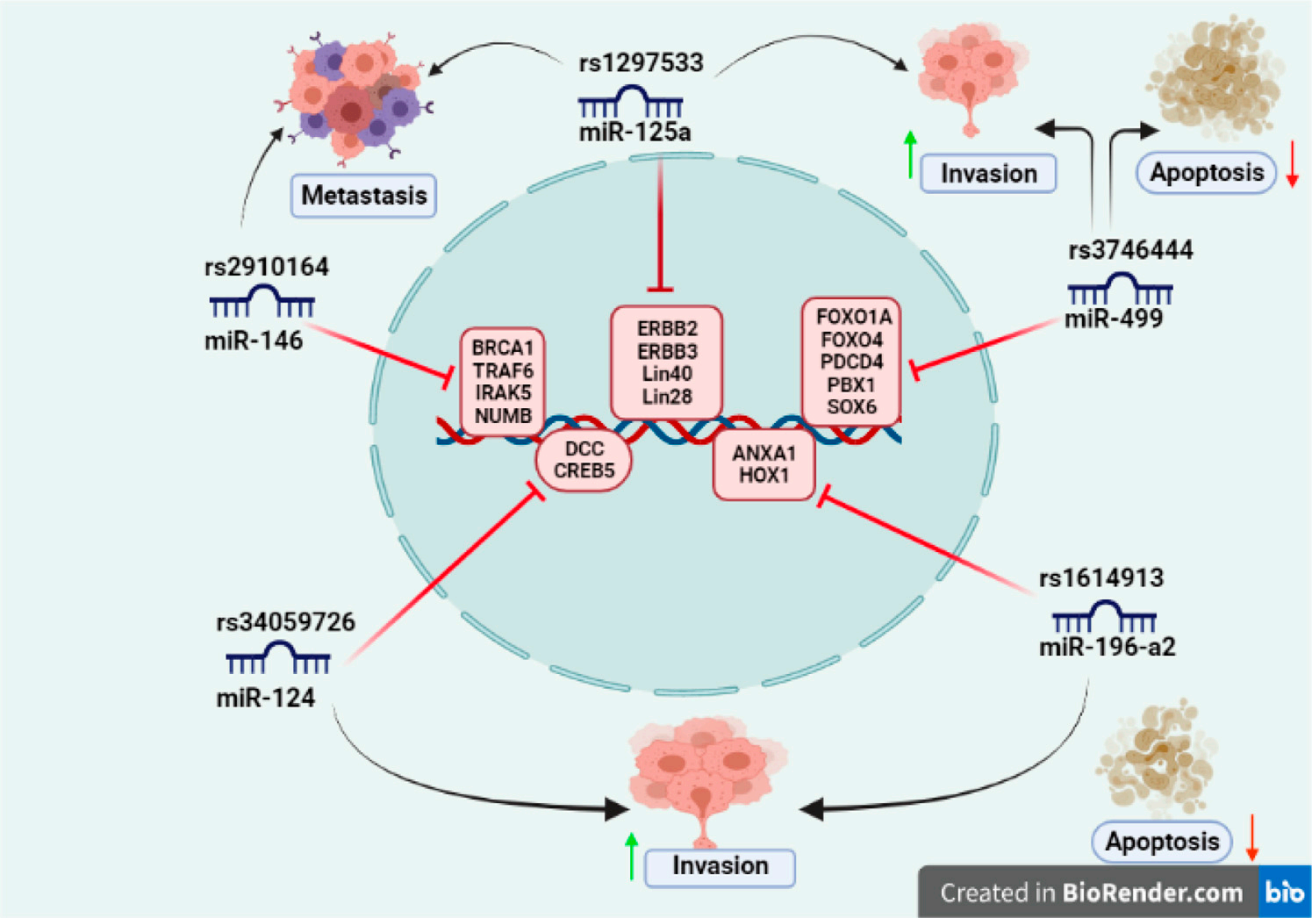
SNPs reported in seed sequence of miRNA involved in cancer: miR-146 (rs2910164) targets include BRCA1, TRAF6, IRAKS, and NUMB gene and associated with cancer metastasis; miR-125a (rs1297533) found to be involved in invasion and metastasis and known targets include ERBB2, ERBB3, lin-40, and lin-28 gene; miR-499 (rs3746444) promotes invasion and inhibits cell apoptosis by targeting FOXO1A, FOXO4, PDCD4, PBX1, and SOX6 gene; miR-124 (rs34059726) has target genes DCC, CREB gene, and enhances invasion; miR-196-a2 (rs1614913) inhibits apoptosis and promotes invasion having target genes ANXA1 and HOX gene.

SNPs in the mature or primary miRNA or seed sequence might affect miRNA processing or binding. Several SNPs present in miRNA main sequences or upstream regulatory regions have been linked to increased cancer risk as well as its prognosis (Duan et al., 2007). In cancer, SNPs reported in seed sequence of various miRNA include rs2910164 in miR-146a; rs3746444 in miR-499; rs12975333 in miR-125a; rs34059726 in miR-124; and rs11614913 in miR-196-a2. Information about SNPs in the seed sequence of miRNAs is summed up in Table 1 and elaborated functional role of these miRNAs in the development of cancer has been depicted in Figure 1.

**TABLE 1:** SNPs reported in seed sequence of miRNA in various cancers.

S. No	miRNA	Gene	SNP reported	Tumor type	Reference
1	miR-379	SEMA3F	rs61991156	Gastric cancer	Cao et al. (2016)
			A>G		
2	miR-627	SEMA3F	rs2620381	ESCC- esophageal squamous cell carcinoma	Cao et al. (2016)
			A>C		
3	miR-499-3p	PBX1, FOXO1A	rs3746444	BC, ALL, colorectal, liver, SCC of head and neck, gallbladder cancer	Xiang et al. (2012); Cao et al. (2016); Ahmad et al. (2019)
			A>G		
4	miR-124	VAMP3, CD164, PTPN12, ITGB1	rs34059726	Lung cancer	Yang et al. (2016); Fawzy et al. (2017)
			G>T		
5	miR-642a	ATP6VOE1	rs78902025	Leiomyoma	Pelletier et al. (2011); Cao et al. (2016)
			T>G		
6	miR-4293	SLC43A2	rs12220909	NSCLC	Malhotra et al. (2019b)
			A>C		
7	miR-146a-3p	BRCA1, TRAF6, IRAK1, NUMB	rs2910164	BC, HCC, PTC, ESCC, colorectal and primary liver cancer	Yekta et al. (2004); Vitale et al. (2011); Xiang et al. (2012); Zhou et al. (2012); Iguchi et al. (2016)
			C>G		
8	MiR-4707	CARD10	rs2273626	Rectal cancer	Shvarts et al. (1996); Erturk et al. (2014)
			C>A		
9	miR-4707	HAUS4	Rs2273626	Rectal cancer	Marine et al. (2006); Erturk et al. (2014)
			C>A		
10	miR-125a	Lin-28, lin-41, ERBB2, ERBB3	rs12975333	Breast cancer	Jin et al. (2008); Tian et al. (2009); Ristori et al. (2017)
			G>A		
11	miR-662	ATPVOE1	rs9745376	Ovarian cancer	Jin et al. (2008)
			G>A/C/T		
12	miR-196-a2	HOX, ANXA1	rs11614913	HCC, BC, NSCLC, bladder, renal, gastric, lung, glioma, head and neck, squamous cancer	Solito et al. (2001); Dou et al. (2010); Nicoloso et al. (2010); Ferracin et al. (2011); Peng and Croce (2016); Malhotra et al. (2019a); Zhao, (2020)
			C>T		
13	miR-585	SLIT3	rs62376935	Pharynx squamous cell carcinoma	Pelletier et al. (2011); Fawzy et al. (2017)
			C>T		
14	miR-605	PRKGI	rs113212828	Ovarian cancer	Pelletier et al. (2011)
			A>G		
15	miR-367-5p	LARP7	rs150161032A > G	Testicular germ cell tumor, leukemia	Strachan et al. (2003); Matijasevic et al. (2008)
16	miR-627	ATP6VOE1	rs2620381	Ovarian cancer, osteoblastoma	Jin et al. (2008); Cao et al. (2016)
			A>C		
17	miR-3161	PTPRJ	rs11382316	Colorectal cancer	Fawzy et al. (2017)
			-/A		

### miRNA-146a

miR-146a is a widely expressed miRNA in mammalian cells. Multiple studies have shown that miR-146a is involved in inflammation, differentiation, and function of adaptive and innate immune cells. miR-146a has been found to be a regulator of cell function and differentiation in innate and adaptive immunity. A subset of human T cells exhibits different expression level of niR-146a. Memory T cells and naive T lymphocytes have different levels of miR-146a expression (Nahand et al., 2020). This miRNA is produced by T-cell receptor activation, and the binding sites of c-ETS and transcription factor nuclear factor-κB (NF-kB) are required for miR-146a transcription in T lymphocyte cells (Curtale et al., 2010; Lu et al., 2010). Some studies observed an association between the NF-kB signaling pathway and miR-146a expression (Rusca and Monticelli, 2011). Taganov et al. (2006) found that LPS stimulation enhanced miR-146a expression in an NF-kB-dependent manner and that miR-146a targeted the IRAK1 and TRAF6 genes (Taganov et al., 2006; Rusca and Monticelli, 2011). After a cell surface receptor (such as TLR4) is activated, a biochemical cascade involving IRAK1 and TRAF6 causes IkBa to be phosphorylated and degraded, resulting in the activation of NF-kB and its nuclear translocation. Furthermore, NF-kB activation causes some genes, such as pri-miR-146a, to be expressed. miR-146a matures on the RISC and contributes to the attenuation of receptor signaling by downregulating TRAF6 and IRAK1. As a result, miR-146a inhibits the signaling pathway leading to NF-B activation (Taganov et al., 2006; Taganov et al., 2007; Labbaye and Testa, 2012).

miR-146a polymorphism, rs2910164, involves a G>C nucleotide alteration on the seed region of miR146a-3p, resulting in G:U pair to a C:U mismatch pairing in the stem of the miR-146a affecting the specificity of mature miR-146a binding to its targets and results in elevated expression of miR-146a (Brincas et al., 2020). Previous studies have established the association of rs2910164 in pre-miR-146a with strong association with breast cancer (BC), hepatocellular carcinoma (HCC), papillary thyroid carcinoma (PTC), esophageal squamous cell carcinoma (ESCC), primary liver cancer, and colorectal cancer (Hu et al., 2009; Vitale et al., 2011; Xiang et al., 2012; Zhou et al., 2012).

A microarray-based expression study carried out in a Chinese population found that miR-146a was significantly upregulated in breast carcinoma tissues compared to normal tissues (Omrani et al., 2014). Its expression level was three times higher in triple negative tumors in comparison with other tumor subgroups. However, this association has been reported to vary in different ethnic groups. The allele C is associated with BC risk in the European population but did not show any association with BC in the Asian population. This discrepancy might be on account of ethnicity, different exposure to carcinogens, or linkage disequilibrium with different causal variants (Brincas et al., 2020).

Molecular targets of miR-146a include BRCA1, TRAF6, IRAK1, and NUMB genes (Brincas et al., 2020). The variant allele of rs2910164 leads to increased levels of mature miR-146a that binds with greater affinity to the BRCA1 gene. Alternatively, rs2910146 might disrupt the well-documented role of miR-146a as a mediator of the pro-apoptotic transcriptional factor NF-κB. Also, the expression levels of miR146a-5p were observed three times higher in triple negative tumors compared to other subgroups of mammary tumors (Brincas et al., 2020). Two other significant targets of miR-146a, TRAF6, and IRAK1 are important adapter molecules downstream of the toll-like and cytokine receptors that have a vital role in signaling cell growth and immune recognition (Omrani et al., 2014). Both the genes have been associated with progression and metastasis. The reduced TRAF6 and IRAK1 levels reduce the activity of NF-kB, a potential inducer of proliferation, survival, angiogenesis, and metastasis (Brincas et al., 2020).

rs2910164 of miR-146a has been reported to induce liver metastasis in colorectal cancer (CRC) via Notch and JAK/STAT signaling pathways. Migratory response of NUMB has been observed in CRC cell lines (RKO, HT29, LoVo). NUMB protein is a negative regulator of Notch signaling, miR-146a activates Notch and JAK/STAT3 signaling through suppression of NUMB protein thereby enhancing the metastatic risk. Further, patients of gastric cancer bearing the altered genotype showed a higher expression of miR-146a than the ones bearing the normal genotype (Iguchi et al., 2016). In addition, this polymorphism has also been reported to increase the risk of PTC in a heterozygous condition (Vitale et al., 2011). The reduction in expression level of miR-146a led to less efficient inhibition of target genes TRAF6 and IRAF1 involved in the Toll-like receptor and cytokine signaling pathways and thereby increase risk of PTC (Slaby et al., 2012).

### miRNA-499

miR-499 is a microRNA that regulates multiple genes and signaling pathways post-transcriptionally, especially in hypoxic-ischemic situations like cancer and myocardial infarction (Wilson et al., 2010; Ando et al., 2014). Wang et al. (2015) observed reduced expression of miR-499-5p disrupted the PI3K/AKT/GSK signaling pathway (Liu et al., 2011). miR-499 functions as a tumor suppressor by decreasing cell proliferation causing apoptosis, which inhibits cancer progression. In addition, it also prevents metastases. FOXO4 and programmed cell death 4 (PDCD4) genes have been reported to be the targets of miR-499 (Liu et al., 2011). PDCD4 is an RNA-binding protein that stops particular mRNAs from being translated (Ohnheiser et al., 2015). PDCD4 modulates several signal transduction pathways and impacts the translation and transcription of many genes as a tumor suppressor (Wang et al., 2013a). It may play a key role in halting cell cycle progression and preventing tumor metastasis by inhibiting cell proliferation (Wei et al., 2012). Wei et al. (2012) demonstrate that PDCD4 may be important in stopping cell cycle progression at a critical checkpoint, limiting cell proliferation and suppressing tumor spread. In ovarian cancer cells, the PI3K-Akt pathway was thought to be involved in the regulation of PDCD4 degradation (Wei et al., 2012).

An SNP rs3746444 (T>C) has been reported in the seed region of mature miR-499 (Chen et al., 2014b). This SNP has been associated with increased susceptibility to various cancers like BC, cervical squamous cell carcinoma, acute lymphoblastic leukemia (ALL), colorectal cancer, liver cancer, gallbladder cancer, lung cancer, gastric cancer, squamous cell carcinoma of the head and neck, and prostate cancer. rs3746444 has been reported to be associated with an elevated risk of BC in the Chinese, German, and Italian populations; gastric cancer in the Japanese population; prostate cancer in the Indian population; cervical squamous cell carcinoma and lung cancer in the Chinese population, and ALL in the Iranian population (Tian et al., 2009; Catucci et al., 2010; Liu et al., 2010; Srivastava et al., 2010; Zhou et al., 2010; George et al., 2011; Okubo et al., 2011; Hasani et al., 2014). In contrast, Asian populations with the T allele of the miR-SNP are thought to have a lower risk of BC whereas Caucasians bearing the same variant allele have been reported at a higher risk of BC (Chen et al., 2014c).

rs3746444 leads to overexpression of miR-499 resulting in its enhanced binding to its target genes including FOXO4, PDCD4, and SOX6 gene (Dai et al., 2016). FOXO transcription factors regulate a variety of physiological activities, including fuel metabolism, oxidative stress response, and redox signaling, cell cycle progression, and apoptosis (Urbánek and Klotz, 2017). FOXO4 is a tumor suppressor protein that has associated with metastasis (Yang et al., 2006; Lee et al., 2009; Zhang et al., 2009). PDCD4 is a well-known tumor suppressor regulating the growing, invading, or metastasis of the tumors. The study reported that prometastatatic action of miR-499 is on account of the suppression of FOXO4 and PDCD4 expression (Liu et al., 2011). PDCD4 inhibits the expression of mitogen-activated protein kinase (MAP4K1) via Jun N-terminal kinase (JNK) pathway. This was established by cDNA microarray analysis of PDCD4-overexpressing in RKO human colon cancer cells (Yang et al., 2006).

rs3746444 has also been reported to regulate the expression level of SOX gene. The anti-apoptosis action of miR-499 (rs3746444 T>C) can be reversed by up-regulating the SOX6 gene (Li et al., 2013a). Deregulation of the SOX gene activates the Wnt/-catenin signaling pathway, which has been linked to cancer development (Yan et al., 2017).

### miRNA-125a

miR-125 plays a role in disease prevention and promotion, especially in cancer and host immunological responses. miR-125 inhibits a variety of genes, including transcription factors, matrix metalloproteinases, Bcl-2 family members, and others, causing aberrant cell proliferation, metastasis, and invasion, as well as carcinomas (Sun et al., 2013). BC, stomach cancer, and medulloblastoma all have lower levels of miR-125a, which promotes disease development. In human medulloblastoma cells, overexpression of miR-125a resulted in cell growth arrest and apoptosis. Furthermore, in stomach cancer cells, identical ectopic expressions inhibited growth. In BC cells, overexpression of miR-125a resulted in decreased anchorage-dependent proliferation. It was discovered that miR-125a modulates these cellular processes through Erbb2 in the context of gastric cancer and BC investigations (Scott et al., 2007; Ferretti et al., 2009).

The polymorphism rs12975333 (G>T) in miR-125a is in the seed sequence at the 8th nucleotide of mature miRNA. The T allele has been shown to inhibit the conversion of pri-miRNA to pre-miRNA precursor and is extremely rare, having been found only once in a panel of 1200 people from various ethnic origins and correlated with an elevated risk of BC in the Belgium population (Peterlongo et al., 2011). The reduced expression of mature miR-125a leads to the overexpression of the target genes.

The known targets of miR-125a like ERBB2 and ERBB3 have previously been reported to be associated with BC tumorigenesis (Morales et al., 2018). ERBB2 encodes the BC marker HER2 and alterations of ERBB2 and ERBB3 have been reported to promote malignancy. For example, ERBB2 overexpression is associated with approximately 25% of all human BC which drives the key aggressive features including cell proliferation, motility, and invasion (Lehmann et al., 2013). Malignant transformation can be induced by deregulation of ERBB2 and ERBB3 alone or in combination. Amplification and overexpression of ERBB2 have been associated with 25% of all human breast tumors. Overexpression of ERBB2 in particular promotes cell survival, proliferation, motility, and invasion, all of which are hallmarks of this aggressive form of human BC (Scott et al., 2007).

### miRNA- 124

miR-124 is one of the most abundant miRNAs in the adult brain and is expressed primarily in the CNS. Mature miR-124 family includes three members, namely miR-124-1, miR-124-2, and miR-124-3. miR-124 has been demonstrated to induce cell differentiation while inhibiting cell proliferation in general (Ristori et al., 2017). Several cancers, including colon, breast, and lung cancers, as well as leukemia and lymphoma, are linked to miR-124 (Pal et al., 2015).

A G>T (rs3405972) has been reported in the seed sequence of miR-124-3 (Gong et al., 2012; Beretta et al., 2017). The major miR-124-3 targets include vesicle-associated membrane protein 3 (VAMP3), sialomucin core protein 24 (CD164), tyrosine-protein phosphatase non-receptor type 12 (PTPN12), neuronal growth regulator 1 (NEGR1), cyclin-dependent kinase 6 (CDK6), integrin Beta 1 (ITGB1), and insulin-like growth factor-binding protein 7 (IGFBP7) (Leong, 2013).

The 3′UTR of oncogene CDK6 is the target of mature miR-124. miR-124 epigenetic masking causes CDK6 activation and subsequent phosphorylation of retinoblastoma (Rb), resulting in cell growth acceleration which is directly involved in brain cancer (Pal et al., 2015). miR-124 expression leads to the downregulation of PTPN12 protein which regulates tyrosine phosphorylation and is implicated in cancer and cellular physiology. As PTPN12 reduces mammary cell proliferation and transformation, the targeting of PTPN12 by miR-124 suppresses its tumor suppressor behavior which promotes the oncogenic shift in breast and lung cancer (Sun et al., 2011). Leong Pei predicted that miR-124-3 with a variant allele targets novel genes DCC (deleted in colorectal cancer) and CREB5 (cyclic AMP-responsive element-binding protein 5) rather than PTPN12 (as predicted by public databases). The variant miR-124-3 is unable to suppress PTPN12 tumor suppressor and may alternatively behave as a tumor suppressor instead of an oncogene in breast or lung cancer (Leong, 2013). Hunt et al. (2011) reported miR-124 reduces oral squamous cell carcinoma (OSCC) invasion by targeting ITGB1, which is responsible for regulating intracellular signaling cascades and tissue homeostasis. Thus, miR-124 has a strong potential to be used as a prognostic marker in OSCC (Sun et al., 2011).

### miRNA-196-a2

miR-196 family of molecules can operate as tumor suppressors. miR-196a, for example, inhibits metastasis in melanoma and BC cells, while miR-196b is downregulated in many types of leukemia cells. Bioinformatics research revealed that miR-196a2 could target multiple genes involved in cell cycle regulation, survival, and apoptosis, all of which could be relevant in GI malignancies. Cell proliferation, migration, invasion, and radio resistance are all functions of miR-196 family molecules’ carcinogenic impacts (Fawzy et al., 2017).

The polymorphic C>T (rs11614913) is in the mature sequence of miR-196a-3p that negatively affects the processing of precursor miRNA to mature and subsequently its capability to regulate its target genes. Variant T allele influences the stability of the secondary structure of miR-196a2 (Wojcicka et al., 2014). This variant has been associated with an increased risk of bladder cancer; renal cancer; gastric cancer; lung cancer, HCC; glioma; head and neck squamous carcinoma; NSCLC and familial BC (Hu et al., 2008; Tian et al., 2009; Dou et al., 2010; Stenholm et al., 2013; Dai et al., 2015; Liu et al., 2018).

C allele impairs mature miRNA expression, resulting in lower levels of mature miR-196a2 (Yekta et al., 2004; Hoffman et al., 2009). In the Chinese population thus miR-SNP-induced decrease in miRNA expression could be used as a predictive biomarker for assessing BC risk (Qi et al., 2015). Other studies, on the other hand, have suggested that in some groups, the rs11614913 polymorphism predicts a lower risk of BC (Dai et al., 2016). A meta-analysis of 16 studies was carried out and observed that Caucasian patients had a lower risk of BC, with no significant influence on total risk (Zhang et al., 2017). It has been found that people with the CC genotype of this SNP were more likely to develop BC (Ferracin et al., 2011; Wang et al., 2013b).

rs11614913 associated with decreased risk of glioma and BC in Chinese populations and it has been found to be associated with reduced risk of cervical cancer in the Indian population (Hu et al., 2009; Dou et al., 2010; Thakur et al., 2019). According to recent studies, miR-196a2 TT genotype was associated with decreased risk for cervical cancer whereas miR-196a2 and CC/CT genotype was associated with higher risk. In another study, it was shown that C allele exhibited association with HCC in the Asian population but not in Caucasians whereas it increased the risk of colorectal, glioma, and prostate cancer in the non-African population compared to the African population (Zhao, 2020).

The potential molecular targets of miR-196a2 include HOX and annexin-A1(ANXA1) genes. ANXA1 was shown to be a key modulator of apoptosis and has since been linked to glucocorticoid activities such as cell proliferation suppression and cell migration control. ANXA1 plays a significant role in membrane trafficking, exocytosis, signal transduction, cell differentiation, and apoptosis, among other biological roles (Luthra et al., 2008). Overexpression of miR-196a2 due to variant rs11614913 leads to the suppression of ANXA1 thereby promoting cell proliferation and suppressing apoptosis (Solito et al., 2001; Luthra et al., 2008; Rahim et al., 2019). ANXA1 exhibits varied expression in different cancers. It is upregulated in glioma and oropharyngeal cancer and downregulated in prostate cancer, esophageal squamous cell, and head and neck squamous carcinoma (Rahim et al., 2019). As an upstream regulator, miR-196a has been found to partially direct the cleavage of the mRNA of the HOX gene clusters. HOX genes were shown to be abnormally expressed in BC, and HOXD10 was found to initiate tumor invasion and metastasis (Yekta et al., 2004).

### SNPs in 3′UTR of miRNA Target Sequence

**FIGURE 2:**
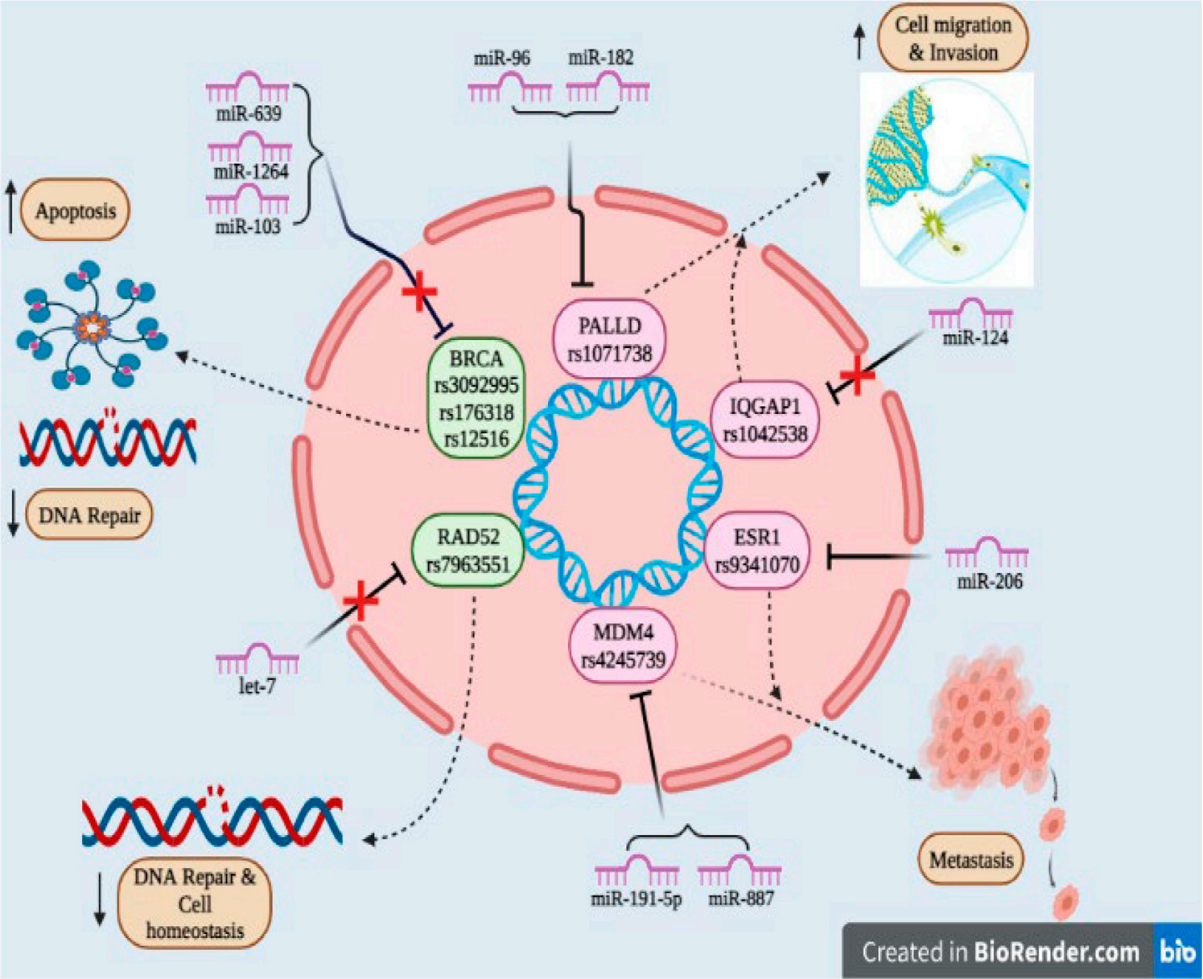
SNPs reported in 3′UTR region of the target gene involved in cancer: Due to SNPs in the BRCA gene (rs3092995, rs176318, rs12516) and RAD52 gene (rs7963551) binding site for miR-639, miR-1264, and miR-103 within BRCA and for let-7 within the RAD52 gene found to be disrupted and as tumor suppressor genes they promote cell apoptosis, decrease the DNA repair mechanism and maintain cell homeostasis; PALLD (rs1071738), IQGAP1 (rs1042538), ESR1 (rs9341070), and MDM4 (rs4245739) genes act as oncogenes and they are responsible for metastasis, cell migration, and invasion.

Several SNPs in miRNA binding sites or miRNA target gene (3′UTR) disrupt the capability to recognize the specific target. This results in dysregulation of target genes due to changes in miRNA and mRNA interactions (Nicoloso et al., 2010; Zheng et al., 2011). The presence of SNPs in the 3′UTR in an oncogene or a tumor regulatory gene might cause changes in gene regulation can shift the balance of cellular homeostasis toward cancer (Zheng et al., 2011). Variations in the 3′UTR of target genes involved in the stress response or DNA repair modify the activity of effector proteins, resulting in changes in the ability to repair damaged DNA and raising the risk of cancer (Cao et al., 2016). SNPs reported in 3′UTR of target genes of specific miRNA includes rs3092995, rs12516, rs8176318 in BRCA1 gene; rs4245739 in MDM4; rs1042538 in IQGAP1; rs7963551 in RAD52; rs9341070 in ESRI; and rs1071738 in PALLD gene. Information about genetic variation (SNPs) reported in 3′UTR of miRNA target gene along with functional outcome implicated in the pathogenesis of cancer have been summed up in Table 2 and Figure 2.

**TABLE 2:** SNPs reported in target site of mi-RNA (mRNA-3′UTR region) in cancer.

S. No	Gene	miRNA	SNP reported	Tumor type	Reference
1	BRCA1	miR-639	rs8176318 G>T	TNBC	Markey (2011); Malhotra et al. (2019a)
		miR-1264	rs12516 C>T		
		miR-103	rs3092995 G>A,C		
		miR-637			
2	CD86	miR-337/200a-5p/184/212	rs17281995 G>C	Colorectal cancer	Moszyńska et al. (2017)
3	CDKN2B	miR-323-5p	rs1063192	Osteosarcoma	Cao et al. (2016); Elfaki et al. (2019)
			G>A,T		
4	DROSHA	miR-27b	rs10719	Bladder cancer	Moszyńska et al. (2017)
			T>C		
5	ESRI	miR-206	rs9341070	Breast cancer	Moszyńska et al. (2017)
			C>T		
6	ESRI	miR-453	rs2747648	Breast cancer	Wynendaele et al. (2010); Shankaran et al. (2020)
			C>T		
7	HIF1A	miR-199a	rs2057482	Pancreatic ductal adenocarcinoma	Moszyńska et al. (2017)
			T>C		
8	INSR	miR-612	rs1051690 G/A	Colorectal cancer	Moszyńska et al. (2017)
9	IQGAP1	miR-124	rs1042538	BC, ovarian, colorectal, glioblastoma, lung, gastric cancer	Stanford et al. (1986); Olefsky (2001); Sommer and Fuqua (2001); Wunderlich et al. (2004); Brekman et al. (2011); Erturk et al. (2014); Malhotra et al. (2019a)
			T>A,G,C		
10	ITGB4	miR-34a	rs743554	Breast cancer	Wynendaele et al. (2010)
			G>A		
11	MDM4	miR-191-5p, miR-887	rs4245739A > C	SCLC, prostate, ovarian, breast, lung, non-Hodgkin’s lymphoma	Balenci et al. (2006); Dong et al. (2006); Moszyńska et al. (2017); Galka-Marciniak et al. (2019)
12	PALLD	miR-96	rs1071738	Breast cancer	Moszyńska et al. (2017); Alipoor et al. (2018)
		miR-182	C>G		
13	PRKD3	miR-145-5p	rs2160395	CRC- colorectal cancer	(Zhuang and Wang, 2017; Abo-Elmatty and Mehanna, 2019)
		miR-27b-3p	C>A,T		
		miR-27a-3p			
14	PSCA	miR-342-5p	rs10216533	GAC-gastric adenocarcinoma	Cao et al. (2016); Wu et al. (2019)
			G>A,C		
15	RAD52	Let-7	rs7963551 (C allele)	Breast cancer increase	Adams et al. (2007); Malhotra et al. (2019a)
16	TGFBR1	miR-628-5p	rs334348	Breast cancer	Malhotra et al. (2019a); Shankaran et al. (2020)
			A>G,T		
17	TP63	miR-140-5p	rs35592567 C>T	Bladder cancer	Moszyńska et al. (2017)

### BRCA 1

BRCA is one of the well-studied genes associated with BC. This gene has a highly conserved 3′UTR of 1381 nucleotides encodes a 1863 amino acid protein and functions as a tumor suppressor gene that regulates various cellular processes including cell cycle checkpoint control, chromatin remodeling, DNA repair, regulation of transcription, protein ubiquitination, and apoptosis (Yang et al., 2016; Ahmad et al., 2019). The 3′UTR region of the BRCA1 gene plays a pivotal role in the localization, stability, and mRNA transport. SNPs in 3′UTR of this gene might alter genes expression and therefore increase the risk of BC. The prevalence of SNPs in 3′UTR of BRCA1 modulating the miRNA binding site that can emerge as a significant biomarker of the disease (Ahmad et al., 2019).

SNPs including rs3092995, rs12516, and rs8176318 are in the 3′UTR of this gene. rs3092995 (G>A, C) is in the sequence of BRCA1 gene that interacts with the seed sequence of miR-103. rs3092995 is strongly associated with increased risk of BC in African American women. The variant alleles of rs8176318 (G>T) and rs12516 (G>A,T) are associated with ovarian and familial BC in Thai women (Pelletier et al., 2011). rs8176318 is located at the region where miR-639 is assumed to bind. Enhanced risk of BC was reported in individuals with GT or GG compared to TT genotype in the Pakistani population (Ahmad et al., 2019). This SNP is also associated with the risk of TNBC and ovarian cancer in an Irish population. The bearer of the rs8176318 variant allele has been linked to an increased risk of BC in menopausal women, as estrogen levels in these women drop after menopause (Malhotra et al., 2019b).

SNP rs12516 in the 3′UTR of BRCA1 gene alters the binding site of miR-1264, affecting its binding affinity and at the same time creating a binding site for other miRNAs including miR- 4278, miR-4704-5p, and miR-637. Binding of these miRNAs to the 3′UTR of BRCA1 has been associated with higher NC risk. This rs12516 present in the 3′UTR of BRCA1 has been reported to be a genetic marker in the Turkish population associated with an increased risk of BC development (Erturk et al., 2014; Malhotra et al., 2019a).

### MDM4

The murine double minute 4 (MDM4) protein was found as a p53-binding protein and has a fundamental amino acid sequence that is very similar to MDM2 (Shvarts et al., 1996). Over half of all human malignancies have a mutation in p53, the most frequently inactivated gene in cancer. p53 functions as a transcriptional factor that transactivates a set of genes involved in multiple cellular processes such as cell cycle arrest, cellular senescence, energy metabolism, and apoptosis in response to various extra- and intracellular stresses such as oncogene activation, DNA damage, and hypoxia. High concentrations of p53 inhibitors can also inactivate p53 signaling. MDM2 and MDM4, two main negative regulators of p53, are substantially responsible for p53 activity suppression (Marine et al., 2006). MDM4 has also been reported to bind with p21 and direct it to proteasomal destruction without ubiquitination. Mdm4-mediated p21 degradation is independent of MDM2, yet it works together with MDM2 to break the G1 cell cycle arrest (Jin et al., 2008). Deletion of MDM4 causes multipolar spindle formation, increased chromosomal loss, higher proliferative potentials, and increased spontaneous tumor transformation in p53-null cells (Matijasevic et al., 2008). Because MDM4 and MDM2 form heterodimers, Mdm4 depletion may stimulate MDM2 interaction with other proteins such as Rb and p21, enhancing carcinogenesis. MDM4 also inhibits E2F1 transactivation by disrupting E2F1-DNA binding or changing the location of the E2F1 transcription complex (Strachan et al., 2003). E2F1 overexpression enhances the G1/S transition in cells and has been linked to cancer development. E2F1 overexpression, on the other hand, causes both p53-dependent and p53-independent apoptosis (Crosby and Almasan, 2004; Wunderlich et al., 2004; Stanelle and Pützer, 2006). These findings point to MDM4 upregulation, which is found in many malignant malignancies (Markey, 2011).

An SNP rs4245739 (A>C) is located within the 3′UTR region of MDM4. The C allele creates a new binding site for three miRNAs, miRNA-191-5p, miR-887, and miR-3669 (Wynendaele et al., 2010; Stegeman et al., 2015; Anwar et al., 2017). The variant allele of this polymorphism has been reported as a risk factor for many cancers including ovarian cancer, lung cancer, prostate cancer, BC, non-Hodgkin’s lymphoma, esophageal cancer, and retinoblastoma (Wynendaele et al., 2010; Zhou et al., 2013; Gao et al., 2015a; Stegeman et al., 2015; Xu et al., 2016; Anwar et al., 2017).

miR-191-5p showed a greater binding affinity with C allele of rs4245739. In a genotype-based mRNA expression analysis, it was found that C allele was associated with decreased risk of ovarian cancer and retinoblastoma in an Asian population (Xu et al., 2016). MDM4 is overexpressed in A allele genotype and enhances the risk of BC and ESCC in a Chinese population (Zhou et al., 2013; Stegeman et al., 2015). The patients bearing homozygous AA allele were found to have 5.5-fold increased risk of tumor-associated mortality and 4.2-fold increased risk of recurrence (Guo et al., 2016). In an *in vitro* study carried out in PC3 cells in the case of C allele bearers, since it binds with miR-191-5p and miR-887. On other hand, A allele is un-targeted, that is, it directly enhances the risk of prostate cancer (Stegeman et al., 2015). Individuals carrying the rs4245739 C allele express low levels of MDM4 resulting in high DNA repair ability mediated by p53 and thus decreased cancer risk (Zhou et al., 2013).

### IQGAP1

IQ-domain GTPase-activating proteins (IQGAPs) are a multi-domain protein family that regulate a variety of cellular processes such as cell adhesion, migration, extracellular signaling, and cytokinesis (Brown and Sacks, 2006). The first of three human IQGAP homologues, IQGAP1 is expressed throughout the body, whereas IQGAP2 and IQGAP3 are mostly found in the liver and intestine, the brain, and the testis (Weissbach et al., 1994; Nojima et al., 2008; Schmidt et al., 2008). IQGAP1 is hypothesized to contribute to the changed cancer cell phenotype by modulating signaling pathways involved in cell proliferation and transformation, cell-cell adhesion weakening cell motility and invasion stimulation (Johnson et al., 2009). Calmodulin, a ubiquitous calcium-binding protein, regulates IQGAP1 function via the IQ motifs, which are common calmodulin-interacting domains present in many proteins. Calmodulin is thought to affect IQGAP1 function by producing a conformational shift that affects IQGAP1-protein interactions and/or IQGAP1 subcellular localization (Briggs and Sacks, 2003). Both ERK and b-catenin-dependent signaling are aided by IQGAP1. IQGAP1 binds to B-Raf, MEK, and ERK leading to the activation of MAPK cascade. Constitutive MAPK pathway activation is a common oncogenic trigger in a variety of cancers, particularly those caused by Ras and B-Raf activating mutations. H-Ras and R-Ras were shown to have no detectable binding whereas active M-Ras had a favorable association (Roy et al., 2005; Nussinov et al., 2018). IQGAP1 is overexpressed in several cancers including ovarian cancer, colorectal cancer, glioblastoma, lung cancer, and gastric cancer (Nabeshima et al., 2002; Nakamura et al., 2005; Balenci et al., 2006; Dong et al., 2006).

miR-124 regulates IQGAP1 through a binding site in its 3′UTR. This target site sequence is disrupted by rs1042538 (T>A,C,G) in the core binding region. The presence of this variation at the miR-124 binding region has been suggested as a possible predictor of BC risk and prognosis. Based on a case-control study carried out in the Chinese population, the TT genotype was associated with a lower BC in comparison AA genotype, depicting that the T allele protects against BC (Zheng et al., 2011).

### RAD52

Radiation sensitive 52 (RAD52) is a DNA strand exchange protein that mediates the DNA-DNA interaction required for complementary DNA strands to anneal during homologous recombination in DNA damage repair in order to maintain cell viability and homeostasis. Recent research has found that RAD52 plays a key function in mammalian cell genomic stability and cancer suppression (Feng et al., 2011; Lok et al., 2013). RAD52 stimulates the creation of nuclear foci, which appears to correspond to DNA repair sites, in response to DNA damage. RAD52 activity increases progressively as cells enter phase S, peaking in the S phase, and then disappearing at the start of G2. Phosphorylation and sumoylation are two post-translational changes that RAD52 can undergo. All these processes appear to work together to control the timing of RAD52 recruitment, as well as its stability and function (Liu et al., 1999; Barlow and Rothstein, 2010; Nogueira et al., 2019). RAD52 has also been shown to have a role in the response to oncogene-induced DNA replication stress (Sotiriou et al., 2016). High levels of RAD52 expression have been reported in tumor cells, particularly in lung squamous cell carcinomas and nasopharyngeal carcinoma tissues (Lieberman et al., 2016).

An SNP (C>A) rs7963551 is in the 3′UTR of RAD52 that is the binding site of let-7 miRNA (Jiang et al., 2013). In Chinese women, this SNP has been linked to an increased risk of BC. The presence of this variation reduces the binding capacity of let-7 to its target regions in the RAD52 3′UTR that has been suggested to boost its expression (Jiang et al., 2013). rs7963551 polymorphism with A allele was found to be strongly related with a lower incidence of SCLC in the Chinese population, according to a study. The functional genetic variant was only substantially associated with SCLC susceptibility among smokers but not with nonsmokers (Han et al., 2015).

### ESR1

The nuclear hormone receptor and oncoprotein estrogen receptor alpha/estrogen receptor 1 (ER/ESR1) is overexpressed in around 70% of breast tumors (Stanford et al., 1986; Sommer and Fuqua, 2001). The ESR1 gene encodes estrogen receptor (ER), which is primarily a nuclear protein that operates as a ligand-dependent transcription factor (ER’s genomic activity) (Olefsky, 2001). In primary human BC and human BC cell lines, MDM2 expression has been reported to be correlated with ER expression. The ER has been postulated to upregulate MDM2 expression (Baunoch et al., 1996; Hori et al., 2002; Brekman et al., 2011). MDM2 also forms a protein complex with ER, making it easier for ER to be ubiquitinated and degraded resulting in a negative feedback loop. However, the ability of ER and MDM4 (another member of the MDM family) to interact and regulate each other’s expression in a comparable way has yet to be established (Liu et al., 2000).

The SNP rs9341070 (C>T) is one of the known polymorphisms located at 3′UTR of ESR1 gene at the binding site of miR-206. This variant influences the binding between miRNA and 3′UTR of ESR1 results in lower expression of ESR1 gene (Brucker et al., 2017). The T allele at 3′UTR allows binding of miR-206 to ESR1 and it is significantly downregulated (Adams et al., 2007). This SNP has been associated with an increased risk of BC (Anwar et al., 2017; Brucker et al., 2017).

### PALLD

Palladin (PALLD), an actin-associated protein whose expression is intimately linked to the pathogenic cell motility properties of aggressive cancer cells, is encoded by the PALLD gene. Palladin expression is higher in invasive and malignant BC cell types than in noninvasive and normal cell lines. Palladin stimulates podosome formation, modulates the actin cytoskeleton via numerous routes, participates in matrix breakdown, and hence it aids in BC spread (Goicoechea et al., 2009; von Nandelstadh et al., 2014).

miR-96 and miR-182 inhibit BC cell migration and invasion by downregulating Palladin protein levels. This mechanism is disturbed by an SNP rs1071738 in the 3′UTR of the PALLD gene. The variation rs1071738 (C>G) is a very normal functional variant of the PALLD gene. The alternate G allele is substantially more prevalent than the ancestral minor C allele. The mRNA target sequence at the 3′UTR of PALLD is entirely complementary to the miR-96 and miR-182 seed areas. The presence of C allele favors the interaction of these miRNAs with the 3′UTR of PALLD. However, the presence of the variant G allele results in a mismatch between the two. miR-96 and miR-182 regulate the expression of PALLD reducing its expression by about 30 and 70%, respectively, in the presence of the normal CC genotype. However, the presence of the G allele leads to abolition of interaction between the two regulatory miRNAs and PALLD, on account of the mismatch between these two miRNAs and seed sequence of PALLD. In normal conditions miR-96 and miR-182 are involved in the prevention of BC metastasis. However, the G allele counteracts this impact (Gilam et al., 2016). The functional significance of rs1071738 has been proved by *in vitro* study carried out by MCF-7 (non-invasive BC cell lines) and Hs578 (highly invasive BC cell line) (Gilam et al., 2016; Moszyńska et al., 2017).

## SNPs in Seed Sequence of miRNA and the 3′UTR of Specific Target Genes in Diabetes

### SNPs in Seed Sequence of miRNA

**FIGURE 3:**
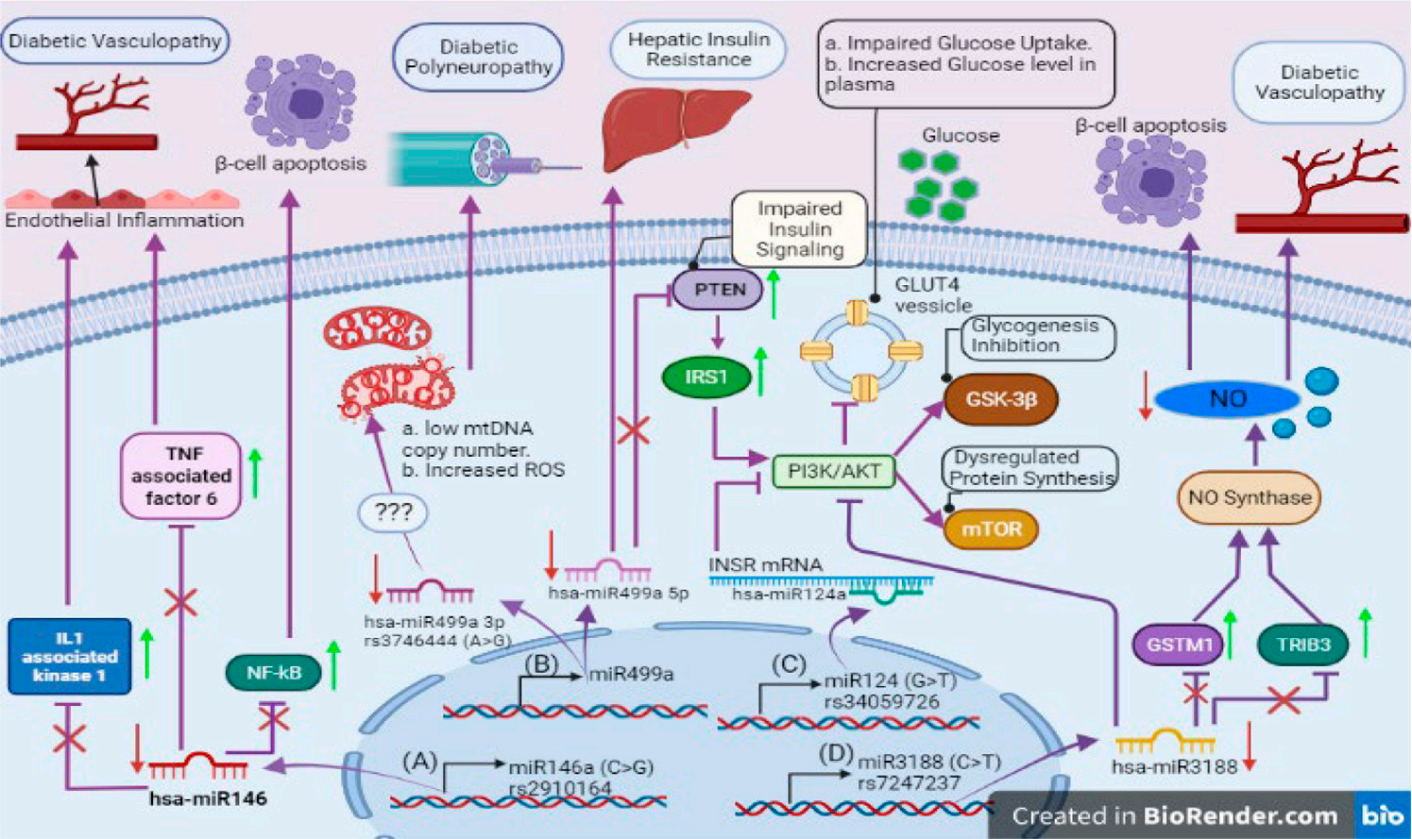
SNPs reported in seed sequence of miRNA involved in diabetes: **(A)** miR146a with SNP rs2910164 (C>G) raises vascular complications caused by upregulation of inflammatory factors (TNF associated factor 6 and IL1 associated kinase 1) in endothelial cells and induces apoptosis in pancreatic β-cells via NF-kB mediated pathway. **(B)** Reduced expression of miR499a due to SNP rs3746444 (A>G) provokes mitochondrial stress, impairs insulin signaling via PTEN mediated pathway, and promotes hepatic insulin resistance. **(C)** The miR124 rs34059726 (G>T) creates complimentary sequence for INSR; causing failure to transport GLUT4 transporter vesicle to outer membrane. This SNP also inhibits glycogenesis process via GSK-3β activation. **(D)** miR-3188 with rs7247237 (C>T) inhibits PI3K/AKT pathway dysregulating protein synthesis. Its target genes overexpression (GSTM1 and TRIB3) curtails nitric oxide pathway which increases vascular complications and induces β-cell apoptosis.

SNPs reported in the seed sequence of miRNA associated with diabetes or its complications including rs3746444 in miR-499a; rs2910164 in miR-146a; rs7247237 in miR-3188; and rs34059726 in miR-124-3p. A detailed information involving SNPs in seed sequence of miRNA associated with diabetes and their functional implications has been given in Table 3 and Figure 3.

**TABLE 3:** SNPs reported in seed sequence of miRNA involved in diabetes.

S. No	miRNA	Target gene	SNP reported	Association with DM	Reference
1	miR-124a	Mtpn1, FOXA3, Sirt1, AKT1	rs531564	Protective role, facilitates glucose metabolism and insulin exocytosis	Wang et al. (2019); Zhao et al. (2013); Hribal et al. (2011)
			G>C		
2	miR146a	NF-kB, TNF associated factor 6, IL1R associated kinase 1	rs2910164 (C>G)	β-cell apoptosis	Zhao et al. (2013); Klöting et al. (2009)
3	miR3188	GSTM1, TRIB3	rs7247237 (C>T)	Impaired insulin signaling and apoptosis of human endothelial cells	Fawcett et al. (2010); Zhao et al. (2013)
4	miR126	PI3K Subunit-2 and SPRED-1	rs4636297 (A>G)	Protective factor against diabetic retinopathy, maintains vascular system integrity	Zhao et al. (2013)
5	miR125a	ENPP1	rs12976445 (T>C)	Regulates IL6R leading to diabetic nephropathy	Fonseca et al. (2005); Bantubungi et al. (2014)
		IL6R			
6	miR375	ADIPOR2	rs6715345 (G>C)	T1DM, T2DM, Insulin resistance syndrome	Mead et al. (2002); Zhao et al. (2013)
7	miR-499	PTEN	rs3746444	Diabetic neuropathy, impaired insulin signaling	Wang et al. (2019)
			A>G		

### miRNA-499a

The diabetic neuropathy including cardiovascular autonomic neuropathy (CAN) and diabetic neuropathy (DPN) have been reported to impact the quality of life in diabetics since these complications have been reported in a large percentage of diabetics. The miR-499a is an antiapoptotic and cardioprotective miRNA (Wang et al., 2011; de Carvalho et al., 2019). It has been reported that polymorphisms in miR499 are involved in perturbed insulin secretion, CAN, and peripheral neuropathy (Ciccacci et al., 2018).

The genetic variation in miR-499a has been associated with the development of diabetic neuropathies. Especially the patients carrying the GG genotype of rs3746444 (A>G) present in the seed region of this miRNA are at higher risk of developing the CAN (Ciccacci et al., 2018). A study carried out in an Italian population investigate the association between mitochondrial DNA (mtDNA) copy number and rs3746444 in DPN patients. A decline in the mtDNA copy number in T2DM patients affecting DPN was observed in comparison with healthy controls (Latini et al., 2020). The increase in the copy number of mtDNA in association with the variant allele has been hypothesized to be on account of mitochondrial fission due to oxidative stress (Ghaedi et al., 2016). Increased ROS and mitochondrial injury might be contributing to nervous system dysfunction (Wang et al., 2011).

Apart from mitochondrial dysfunction, dyslipidemia was also observed in patients. Dyslipidemia plays a significant role in the pathogenesis of DN, synergistically with hyperglycemia (Vincent et al., 2009). An excess of long-chain fatty acids in T2D can lead to an accumulation of acetyl-CoA, as a product of mitochondrial beta-oxidation (Fan et al., 2020). miR499a-5p over-expression can enhance the glycogen level and improve insulin signaling by PTEN inhibition. Reduced miR-499-5p level is observed in hepatic insulin resistance (Wang et al., 2015a). miR-499-5p is involved in the signaling pathway of IRS1/PI3K/AKT and in particular miR-499-5p targets PTEN, which is an important regulator of the insulin signaling pathway (Peyrou et al., 2015). Therefore, unstable secondary structure with GG genotype reduces miR-499-5p levels, as a consequence an increase in PTEN impairs the insulin signaling.

### miRNA-146a

The key biological role of miR146a is as immunosuppressive modulator which regulates inflammatory response. It downregulates innate immune response by suppressing expression of IRAK1 and TRAF2, decreasing NFkB activity (Gholami et al., 2020). Therefore, it functions as a negative regulator of NFkB and its inflammatory cascade and promotes apoptosis and inhibits migratory capacity by negative regulation of EGFR signaling pathway (Chen et al., 2013; Park et al., 2015).

SNP rs2910164 C>G is present within the seed sequence of miR146a which reduces its expression (Alipoor et al., 2018). This SNP plays a significant role in the pathogenesis of diabetes by participating in β-cell metabolism, proliferation, and death. The suppressed expression of miR146a enhances the activity of NFkB inflammatory events and induction of β-cell apoptosis responsible for diabetes and related complications (Elfaki et al., 2019). The potential targets of miR146a include TNF associated factor 6 and IL-1 receptor associated kinase 1 which regulate endothelial inflammation. The C>G transition causes overexpression of these target mRNAs resulting in T2DM (Shankaran et al., 2020). This polymorphism also increases the incidence of preeclampsia in gestational diabetes mellitus (GDM) (Abo-Elmatty and Mehanna, 2019).

In a study involving the Chinese population, rs2910164 was associated with an increased risk of T2DM. In some other studies, this SNP is also responsible for risk like diabetic nephropathy in T1DM patients and diabetic macular edema in T2DM patients of Caucasian population. Further it is also associated with diabetic polyneuropathy and GDM in the Italian and Egyptian populations (Zhuang and Wang, 2017).

### miRNA-3188

miR-3188 is involved in regulation of the mTOR-P-PI3k/AkT pathway and has been reported to affect the pathogenesis of diabetic complications. It is one of the miRNAs discovered quite early.

rs7247237 (C>T) is considered to be located in the seed sequence of miR-3188 and has been associated with T2DM in the Chinese population. *In vitro* studies on HUVEC cell lines showed that the C allele expression was five times higher than T allele suggesting that C>T transition reduces its level which results in the overexpression of its targets; GSTM1 (glutathione S-transferase M1) and Trib3 (Tribbles pseudokinase3). This in turn reduces nitric oxide (NO) production in the endothelial cells through inhibition of endothelial NO synthase. There is also evidence that the overexpression of TRIB3 is associated with apoptosis in human endothelial cells, which could probably have an important role in the progression and pathogenesis of vascular complications in diabetes. As according to a study, miR3188 regulating mTOR and PI3K/AKT pathway involved in insulin signaling in endothelial cell; its reduced expression on account of the presence of rs7247237 resulting in T2DM (Wang et al., 2017; Wu et al., 2019).

RhoA/ROCK is another downregulated pathway by miR-3188 is RhoA/ROCK pathway via targeting ETS transcription factor ELK4. Elk4 is involved in various cancers and atherosclerosis. Its potential role via RhoA/ROCK pathway needs to be further elucidated (Li et al., 2017). The potential therapeutic value of miR-3188 could be further explored to mitigate the effect of pathogenic SNP (Wang et al., 2019)

### miR-124-3p

The miR-124 is highly expressed in the brain and involved in epigenetic regulation of neurogenesis (Coolen et al., 2015). However, the *in vitro* studies miR124 overexpression of miR-124 in MIN6 pseudoislet cells caused impaired glucose induced secretion of insulin. Its silencing in MIN6 pseudoislet cells resulted in upregulation of its target genes FOXA2, Mtpn, Flot2, AKT3, Sirt1, and NeuroD1. All these targets are involved in normal Beta-cell functioning (Sebastiani et al., 2015). A study carried out in a mouse model demonstrated that miR-124 mediates triglyceride accumulation in the liver induced by high fat diet by directly targeting tribbles pseudokinase 3 (TRB3) and enhancing AKT signaling (Liu et al., 2016). An SNP rs34059726 located in the seed region of miR-124-3p, curated in PolymiRTS database is predicted to target insulin receptor transcript (INSR) (Gong et al., 2012). INSR belongs to tyrosine kinase receptor family which mediates insulin signaling via PI3K/AKT pathway. This pathway is responsible for maintaining glucose homeostasis, proliferation, and differentiation and inhibition of apoptosis (Chen et al., 2019). Down regulation of INSR via miR-124-3p leads to dysregulation of glucose uptake due to inhibition of GLUT4 vesicle transport to membrane and glucose transfer into cells (Jaakson et al., 2018). Lower PI3K/AKT signaling stimulates GSK-3β and inhibits glycogenesis. Failure of AKT to inhibit proapoptotic protein expression leads to apoptosis of the cells.

### SNPs in 3′UTR of miRNA Target Sequence

**FIGURE 4:**
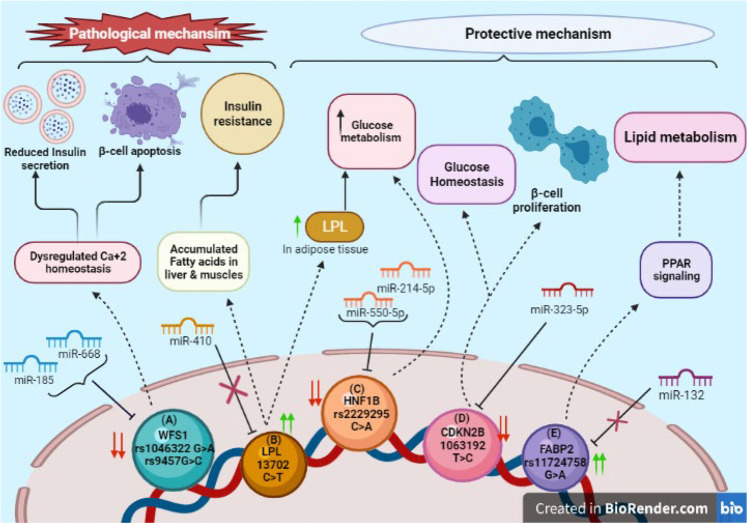
SNPs in 3′UTR of target genes associated with diabetes: **(A)** Downregulation of WFS1 due to SNPs rs1046322 (G>A) and rs9457 (G>C) via miRNAs miR185 and miR668 induces β-cell apoptosis and declines insulin secretion due to ER Ca^+2^ stress. **(B)** SNP rs13702 (C>T) in LPL gene disrupts binding site for miR410. Its overexpression has pathological impact on liver and muscles leading to insulin resistance; whereas in adipose tissue it increases glucose metabolism. **(C)** SNP rs2229295 (C>A) of HNF1B creates new binding site for miR214-5p and miR550-5p, increasing glucose metabolism. **(D)** CDKN2B’s downregulation due to SNP rs1063192 (T>C) by miR323-5p maintains glucose homeostasis and promotes proliferation of β-cells. **(E)** Over-expression of FABP2 due to SNP rs11724758 (G>A) activates PPAR signaling pathway which in-turn leads to lipid metabolism.

The SNPs in 3′UTR of miRNA target genes reported in diabetes include rs11724758 in FABP-2; rs1046322 in WFS-1; rs2229295 in HNF1B; rs1063192 in CDKN2B; and rs13702 in LPL genes. Further details of other SNPs in 3′UTR of miRNA target genes implicated in pathogenesis of diabetes have been summed up in Table 4 and the functional role in Figure 4.

**TABLE 4:** SNPs reported in 3′UTR of miRNA target genes in diabetes.

S. No	Target gene	miRNA	SNP reported	DM association	Ref
1	HNF1B	miR-214 5p, miR-550a-5p	rs2229295 C>A	Susceptibility to T2DM	Xiang et al. (2012)
2	SLC30A8	miR- 183	rs3802177 G>A	T2DM	Grillari and Grillari-Voglauer (2010)
3	WFS1	miR-668	rs1046322	T2DM	Martin et al. (2007)
			G>A		
4	NLRP3	miR-223	rs10754558	Protective against T1DM, increased risk of T2DM via insulin resistance	Scutt et al. (2020)
			C>G		
5	WFS1	miR-185	rs9457 G>C	T2DM	Liu et al. (2014)
6	CDKN2A/B	miR- 323b-5p	rs1063192	Gestational DM	Jeon et al. (2013)
			CC		
7	ENPP1	miR-9	rs7754586	T2DM, end-stage renal disease	Marzec et al. (2007); Marczak et al. (2012)
		miR- 125 a/b	A>C		
8	ENPP1	miR-9, miR- 125 a/b	rs7754561	Insulin resistance and hypertriglyceridemia. Diabetic retinopathy in T2DM.	Thumser et al. (2014)
			A>G		
9	PYY	miR-663	rs162431	T2DM	von Otter et al. (2014)
			G>A		
10	LPL	miR-410	rs13702	T2DM	Schmidt et al. (2000)
			T>C		
11	PIK3RI	miR-29 a/b/c	rs3756668	Insulin resistance, T2DM	Sussan et al. (2008)
			G/G		
12	INSR1	Let-7a, miR27a	rs3745551	T2DM	Sussan et al. (2008)
			G/G		
13	INSR1	miR-106	rs1366600	GDM, T2DM	Tan et al. (2007); Shimoyama et al. (2014)
			T>C		
14	SLC30A8	miR-181a-2-3p	rs2466293	Impaired glucose regulation, reduced β cell function, GDM, T2DM	von Otter et al. (2014)
			T>C		
15	RNLS	miR-96	rs1048956	Affects insulin exocytosis, T2DM	Tan et al. (2007)
			A>G		
16	GSTA4	miR-200a	rs405729	Glucose stimulated insulin secretion	Tan et al. (2007)
			G>A		
17	TRIB3	miR-132	rs2295491	Obesity related impaired insulin secretion	Tan et al. (2007)
			G>A		
18	PRKCE	miR-410	rs41281467	Regulation of insulin secretion	Tan et al. (2007)
			C>T		
19	ACSL1	miR-34a	rs2292899	T2DM	Tan et al. (2007)
			G>A		
20	PDP2	miR-9	rs17767794	Negative control on insulin release	Tan et al. (2007)
			G>C		
21	SLC37A2	miR-9	rs3824926	Negative control on insulin release	Tan et al. (2007)
			T>C		
22	INSR1	miR-20b	rs1366600	T2DM	Tan et al. (2007)
			T>C		
23	FABP2	miR-132	rs11724758 G>A	Impaired insulin sensitivity	Tan et al. (2007)
24	IL10	miR-523	rs6687786	T1DM	Scutt et al. (2020)
			G>A		
25	CTLA4	miR-302a	rs13384548	T1DM	Scutt et al. (2020)
			G>A		
26	VDR	miR125b	rs3847987 C>A	T1DM	Michan and Sinclair (2007) miRdSNP
27	ESR1	miR-122	rs9341070	T2DM and breast cancer	North et al. (2003) miRdSNP
28	SLC15A4	miR-124	rs3765108	T2DM	Vaquero et al. (2006)
29	PPAR-δ	miR-1827	rs3734254	T2DM	Inoue et al. (2007)
30	ADIPOR2	miR-124, miR-375	rs1044471	Insulin resistance, T2DM	de Oliveira et al. (2012), miRdSNP
31	ADIPOR2	miR-1197	rs12342	Insulin resistance syndrome, T2DM	de Oliveira et al. (2012), miRdSNP
		miR-375			
32	LPIN2	miR-1227, miR-27a	rs3745012	T2DM	Crocco et al. (2015) miRdSNP
			G>A,C		
33	IL7R	miR-135b-5p, miR-135a-5p	rs6897932	T1DM	Sebastiani et al. (2015)
34	VPS26A	miR-381-3p	rs1802295	T2DM	Sebastiani et al. (2015)
35	HMG20A	miR-134-5p and miR-494-3p	rs7119	Impaired β-cell function	Sebastiani et al. (2015)
			C>T		
36	MAPK1	miR7-5p	rs12158121	β–cell proliferation by regulating mTOR pathway	Sebastiani et al. (2015)
			A>C		
37	LIN28A	Let-7a	rs3811463 T>C	GDM	Hiratsuka et al. (2003)
38	LIN28A	miR-125a	rs3811463 T>C	T2DM	Hiratsuka et al. (2003)
39	PAX4	miR-125a, miR-223	rs712699	Influences β-cell differentiation and survival	Li et al. (2013b)
			G>A		
40	KCNB1	miR- 448, miR-214, miR-153	rs1051295	Β-cell compensatory secretory function and reduced insulin sensitivity	(Li et al., 2013b) miRdSNP
			T>C		

### FABP-2

Fatty acid binding protein-2 belongs to ubiquitous lipid chaperones family is expressed in intestines which regulate fat absorption by intracellular trafficking of long chain fatty acids, eicosanoids, and other lipids (Haunerland and Spener, 2004). Its dysregulation has been associated with non-alcoholic hepatic liver disease and obesity (Thumser et al., 2014). Around 20–30% T2DM patients have renal impairment and FABP 2 is a novel biomarker for diabetic nephropathy (Tsai et al., 2020). FABP2 has been associated with insulin resistance mechanisms, indicating its essential role in protection against T2DM (Baier et al., 1995).

The 3′UTR polymorphism of rs11724758 (G>A) in FABP2 gene causes loss of binding site for miR-132. The miR-132 plays a significant role in adipose tissue dysfunction and obesity associated diabetes (Klöting et al., 2009). The AA genotype of FABP2 has been associated with decreased risk of T2DM compared to GG genotype (Zhao et al., 2013). Therefore, G>A transition functions as a protective factor against T2DM. FABP2 is involved in intracellular fatty acid transportation and fat absorption via PPAR signaling (Zhao et al., 2013).

### WFS-1

Wolframin or WFS1 gene encodes for endoplasmic reticulum trans-membrane protein highly expressed in brain, pancreas, and heart (Hofmann et al., 2003). Mutation in this gene leads to a metabolic condition known as Wolfram Syndrome inherited in an autosomal recessive manner (Harel et al., 2015). Two SNPs within 3′UTR of WFS1—rs1046322 and rs9457—have been reported to be as risk factors for T1DM and T2DM, respectively (Fawcett et al., 2010; Kovacs-Nagy et al., 2013; Elfaki et al., 2019). WFS1 is an ER transmembrane protein highly expressed in pancreas and insulinoma β-cell lines. It plays a significant role in maintaining Ca+2 ER homeostasis (Hofmann et al., 2003). In T2DM, it indicated that glucose induced insulin secretion has been found to increase WFS1 expression. The increased insulin production caused by insulin resistance in T2DM leads to chronic ER stress contributing to the death of β-cells by apoptosis. It was demonstrated that glucose induced insulin secretion leads to increased WFS1 expression in wild-type mice, whereas ER stress and β-cell dysfunction can be observed in WFS1 knock-out animals (Fonseca et al., 2005). *In vitro* interaction between WFS1 3′ UTR and miR-668 signified the rs1046322 influenced the affinity of miR-668 to WFS1 mRNA. In an *in vitro* luciferase assay it was observed that variation in 3′utr of WFS1 gene rs1046322 “A” and rs9457 “C” is sensitive to both miR-185 and miR-668, although the effect of miR-185 seemed to be stronger (Elek et al., 2015). miR-185 was reported to be strongly associated with diabetes mellitus via its targets SOCS3 and WFS1 (Bao et al., 2015; Elek et al., 2015). They showed that these different pathways can be in the background of the same phenotype, as miR-185 is suggested to be related to diabetes mellitus via WFS1 target (Bao et al., 2015).

### HNF1B

HNF1B encodes for HNF1β (hepatocyte nuclear factor 1-β) homeodomain containing transcription factor expressed in pancreas, liver, and kidney (Coffinier et al., 1999). It regulates the critical function of pancreatic development, glucose metabolism, and hepatic insulin activity (Goda et al., 2015). It is the most common transcription factor associated with monogenic diabetes leading to young adult onset of T1DM with dominant inheritance patterns in familial cases. An SNP rs2229295 (C>A) within 3′-UTR of HNF1β acts as a protective factor against T2DM (Moszynska et al., 2017). In silico analysis revealed that rs2229295 in 3′UTR of HNF1β creates the binding site for miR-214-5p and miR-550-5p. The A allele of HNF1β is responsible for post-transcriptional regulation by miR214-5p and miR-550-5p. Therefore, due to this variation, expression of HNF1β is downregulated and thereby it acts as a protective factor against T2DM (Goda et al., 2015).

### CDKN2B

CDKN2A/B highly expressed in pancreas is considered as a strong determinant of diabetes mellitus. The SNP rs1063192 T>C located within 3′UTR region of CDKN2A/B is associated with increased risk of GDM in pregnant Chinese Han women population (Wang et al., 2015b). The tumor-suppressor products of CDKN2A/B, p15INK4b, and p16INK4a inhibit important CDKs, i.e., CDK4 and CDK6, essential for β-cell proliferation and regeneration (Krishnamurthy et al., 2006). The T>C transition creates complimentary sequence of miR-323-5p which reduces the expression of CDKN2A/B (Hribal et al., 2011). The decreased expression of CDKN2A/B due to rs1063192 T>C results in reduced inhibition of CDK6 by p15INK4b and facilitates β-cell proliferation, lowering DM risk. On the other hand, increased expression of p15INK4b regulates glucose homeostasis. It can be speculated that miR-323-5p may also regulate other crucial genes responsible for β-cell hyperplasia and insulin signaling. Moreover, duality of p15INK4b in glucose homeostasis and deficiency of p16INK4a inducing *in vivo* hepatic glucose production via PKA-CREBPGC1a pathway possibly explains its role in GDM (Bantubungi et al., 2014).

### LPL

Lipoprotein lipase enzyme is involved in hydrolysis of low-density lipoproteins and circulating chylomicrons into non-esterified fats which can be absorbed by the tissues. Disturbance in this conversion could lead to various abnormalities such as Alzheimer’s, dyslipidemia, and diabetes (Mead et al., 2002). In liver and muscle tissues, the free fatty acid generated by LPL activity gets accumulated, leading to insulin resistance (Kim et al., 2001). The SNP rs13702 C>T in 3′UTR of LPL mRNA is located within the seed recognizing region of miR-410 (Hatefi et al., 2018). This C>T transition disrupts the binding site of miR-410 which leads to increased expression of LPL (Richardson et al., 2013). However, in adipose tissue, LPL increases glucose metabolism and insulin tolerance (Walton et al., 2015). Knockdown studies in MIN6 cells indicated decreased ability of glucose induced insulin secretion. In the Iranian population, the T allele of rs13702 showed protective association whereas C allele was found to be a risk factor against T2DM (Hatefi et al., 2018).

## SNPs in the Seed Sequence of miRNA and Target Genes Associated with Various Diseases

SNPs within seed sequence of miRNA and their target genes have been also implicated in the development of various human diseases like Parkinson disease, asthma, periodontal disease, neurodegenerative disease, cardiovascular disease, and kidney and liver diseases (Martin et al., 2007; Tan et al., 2007; Rademakers et al., 2008; Wang et al., 2008; Schaefer et al., 2010; Bruno et al., 2012). The presence of SNPs in miRNA seed regions has a major impact on miRNA target loss and gain (generates a new repertoire of target genes), resulting in a considerable change in miRNA biological function (Xu et al., 2013; Zhang et al., 2019).

Among human diseases, ischemic stroke is one of the complicated diseases that consist of a variety of conditions with different hereditary and environmental risk factors. miRNAs played a role in a variety of physiopathological processes, and frequent SNPs in pre-miRNAs have been linked to disease vulnerability in humans (Liu et al., 2014). According to a case-control study, SNP (A>G) rs3746444 located within seed sequence of miR-499 may be significantly linked with higher risk of ischemic stroke in the Chinese community (Liu et al., 2014). miR-499 has been associated with ischemia condition, apoptosis, and cell death in anoxia via knockdown or calcineurin over-expression, inhibiting Drp1 dephosphorylation and mitochondrial fragmentation caused by anoxia (Wang et al., 2011). The rs3746444 polymorphism changed the stem structure of the miR-499 precursor from an A:U pair to a G:U mismatch, which changed the function or expression of mature miR-499, as well as the regulation of target mRNAs, influencing the risk of ischemic stroke (Hu et al., 2009; Xiang et al., 2012). Its targets includes peptyl arginine deiminase type 4, regulatory factor X4, IL-2, IL-2 receptor B (IL-2R), IL-6, IL-17 receptor B (IL-17RB), IL-18 receptor (IL-18R), IL-21, IL-23a, and B and T lymphocyte attenuator (Yang et al., 2012). miR-499/rs3746444 bound to its mentioned targets and can influence inflammation, fibrinogen, and CRP formation. Higher plasma CRP levels can raise blood pressure, BMI, insulin resistance, and lipids making CRP one of the common causes of cerebral ischemia (Yang et al., 2012; Jeon et al., 2013). Increased CRP, inflammation, and fibrinogen in the allele G and carried G genotypes of rs3746444 A/G may play a predisposing role in the development of ischemic stroke (Liu et al., 2014).

SNP in the 3′UTR of mRNA/target gene might disrupt or create the binding sites for miRNA. The renin-angiotensin system (RAS) is a key player in blood pressure regulation and is thought to be a contributing element in the development of hypertension (Laragh et al., 1991). Angiotensin II is a key player in the RAS pathway, inducing vasoconstriction, salt retention, and water retention, and is closely linked to the inflammatory, thrombotic, and fibrotic factors. Angiotensin II receptor type 1 (AGTR1) and type 2 (AGTR2) mediate these effects both directly and indirectly (AGTR2). AGTR1 is mostly found in vascular smooth muscle cells, as well as the heart, adrenal gland, and kidney (Oparil and Weber, 2000). A study on miR-155 and SNPs in the angiotensin II receptor, type 1 gene has been conducted by Sethupathy et al. (2007). They discovered that miRNA miR-155 could bind to the A allele of the SNP rs5186 (A>C) in the 3′UTR of the AGTR1 mRNA more efficiently than the C allele (which is more common in essential hypertension) (Sethupathy et al., 2007). In persons with the A allele, the binding of miR-155 has the capacity to reduce the level of AGTR1 mRNA and hence cause the pressor effect of Angiotensin II. Protein levels of AGTR1 in untreated essential hypertension patients homozygous for the C allele of rs5186 were also favorably linked with systolic and diastolic blood pressure. The expression levels of miR-155 were also negatively linked with AGTR1 protein levels, and miRNA levels were lower in those with the CC genotype that is directly associated with hypertensions (Ceolotto et al., 2011). Tables 5 and 6 give a brief glimpse of SNPs reported in miRNAs or target gene sequences in various diseases.

**TABLE 5:** SNPs reported in seed sequence of miRNA involve in various disease.

S.No	miRNA	Gene	SNP reported	Disease	Reference
1	miR-1304	SEMA3F	rs79759099	Pulmonary valve disease, pulmonary valve stenosis	Cao et al. (2016)
			A>G		
2	miR-662	ATP6VOE1	rs9745376	SLE	Cao et al. (2016)
			G>A/C/T		
3	miR-96-3p	-	rs546098287	Non-syndromic hearing loss	Malhotra et al. (2019b)
			A>G		
4	miR-548	NS1ABP, MAPK, CDK13	rs515924	Influenza virus infection	Kim et al. (2011); Erturk et al. (2014)
			A>G		
5	miR-122		rs41292412	AMD-age related degeneration	Erturk et al. (2014); Fawzy et al. (2017)
			C>T		
6	miR-431	RTL1	rs12884005	Autism	Hulf et al. (2011); Fawzy et al. (2017)
			A>G		
7	miR-3161	PTPRJ	rs11382316	Androgen insensitivity syndrome	Fawzy et al. (2017)
			-/A		
8	miR-499-3p	BCL2	rs3746444	Ischemic stroke	Cao et al. (2016)
			A>G		
9	miR-3618	DGCR8	rs12159555	Digeorge syndrome	Pelletier et al. (2011); Fawzy et al. (2017)
			C>G		
10	miR-4284	STX1A	rs11973069 C>T	Arteriosclerosis obliterons and pediatric ulcerative colitis	Pelletier et al. (2011)
11	miR-221		rs113054794	Crohn’s disease	Gao et al. (2015b); Fawzy et al. (2017)
			A>C		

**TABLE 6:** SNPs in 3′UTR in target gene of miRNA involved in various disease.

S. No	Gene	miRNA	SNP reported	Disease	Reference
1	APOC3	miR-4271	rs4225	Coronary heart disease	Moszyńska et al. (2017)
			G>T		
2	APOA5	miR-485-5p	rs2266788 G>A	Hyper-triglyceridemia	Moszyńska et al. (2017)
3	PLIN4	miR-522	rs8887	Antropometrics (Obesity related)	Moszyńska et al. (2017)
			T>C,G		
4	FXN	miR-124-3p	rs1145043	Friedrich’s atria FRDA	Moszyńska et al. (2017)
			G>T		
5	SNCA	miR-34b	rs10024743	Parkinson’s disease	Moszyńska et al. (2017)
			T>G		
6	EFNB2	miR-137	rs550067317	Schizophrenia	Moszyńska et al. (2017)
			A>C		
7	FGF20	miR-433	rs12720208	Parkinson’s disease	Cao et al. (2016); Moszyńska et al. (2017)
			C/T		
8	AGTR1	miR-155	rs5186	Hypertension	Jin et al. (2008); Cao et al. (2016); Moszyńska et al. (2017)
			A>C	Renal disease	
9	DHFR	miR-24	rs34764978 C>T	Methotrexate resistance	Moszyńska et al. (2017)
10	HLA-G	miR-48a	rs1063320 C>G	Childhood asthma	Moszyńska et al. (2017)
		miR-152			
11	HTR1B	miR-96	rs13212041 A/G	Arson/property damage	Moszyńska et al. (2017)
12	HTR3E	miR-510	rs56109847 G>A	Diarrhea irritable bowel syndrome	Moszyńska et al. (2017)
13	TGFB1	miR-187	rs1982073 (rs1800470)	Frozen shoulder development	Wynendaele et al. (2010); Hoss et al. (2014)
			G>A,C		
14	MMP9	miR-491-5p	rs1056628	SLE, Early neurologic deterioration	Wynendaele et al. (2010); Sondermeijer et al. (2011)
			A>C		
15	HTR3E	miR-510	rs62625044	Irritable bowel syndrome	Usiello et al. (2000); Cao et al. (2016)
			G>A rs56109847		
16	PFAS	miR-149-3p	rs1132554	Alcohol related neurodevelopment disorder	Cao et al. (2016)
			C>T		
17	TRIB2	miR-877-5p	rs1057001	Obesity	Olefsky (2001); Cao et al. (2016)
			T>A		
18	TMCO1	miR-296-3p	rs6660601	Skeletal anomalies, mental retardation syndrome	Cao et al. (2016)
			C>T		
19	SRSF3	miR-7f-2-3p	rs7344	POAG- primary open angle glaucoma	Crocco et al. (2015); Cao et al. (2016)
			T>C		
20	CAV2	miR-244-5p	rs1052990	POAG	Cao et al. (2016)
			T>C,G		
21	AAGAB	miR-329-5p	rs1050285	Punctuate palmoplantar keratoderma type1	Cao et al. (2016)
			T>C		
22	ABO	miR-855-3p	rs8176751	Hematological phenotype	Cao et al. (2016)
			C>A,T		
23	MTPN	Let-7/miR-98	rs17168525	Cardiac hypertrophy	Jin et al. (2008); Wang et al. (2013a)
			G>A		
24	MS4A6A	miR-382-3p	rs610932	LOAD- Late onset Alzheimer disease	Bäckman et al. (2006); Erturk et al. (2014)
			T>G		
25	TCF-21	miR-224-5p	rs12190287 C>G,T	Acute Coronary syndrome	Noble (2003); Erturk et al. (2014)
26	POCRID	miR-425-3p	rs7097	DLBCL	Abo-Elmatty and Mehanna (2019)
		miR-5444a/miR-507	C>T	Diffuse large B-cell lymphoma	
27	PRKD3	miR-329-3p	rs8243	Polycystic kidney and liver disease	Abo-Elmatty and Mehanna (2019)
		mir495-3p	C>A		
28	ZNF155	miR-708-5p	rs442220	Herpes simplex virus 1	Abo-Elmatty and Mehanna (2019)
		miR-28-5p	G>A,C		

### SNPs Role in Aging

Human aging is a complicated process that has been related to dysregulation of a variety of cellular and molecular processes, including telomere shortening, altered DNA damage response, protein homeostasis loss, cellular senescence, and mitochondrial failure. These cellular and molecular processes can result in a wide range of illnesses, including cancer, cardiovascular disease, and neurological disease, as well as an increased chance of death (Huan et al., 2018). The study of the mechanics of the aging process could also benefit from the determination of an individual’s SNPs. A comparison of the DNA sequences of healthy young individuals with the DNA sequences of healthy, extremely elderly people could reveal genes that play a big role in determining how long people live. Animal models had already been researched, and specific genes, such as DNA repair genes, had also been studied because of the role of repair processes in aging (Schmidt et al., 2000; Ruttan and Glickman, 2002). In recent years, it has also been suggested that post-transcriptional control by miRNAs may play a role in the phenotypic changes seen throughout aging by epigenetically modifying the expression of important regulatory proteins (Grillari and Grillari-Voglauer, 2010; Liu et al., 2012).

Human aging is linked to increased susceptibility to adverse drug reactions (ADRs), multimorbidity, and frailty, however, the intensity and age at which people become ill varies greatly. Identifying genetic indicators for this phenotype’s higher risk might aid in the stratification of individuals who would benefit from specialized intervention. Nuclear factor (erythroid-derived 2)-like 2 (Nrf2) controls the expression of enzymes involved in drug metabolism, as well as the cell’s response to stresses. In animal aging models, its expression has been demonstrated to diminish (Scutt et al., 2020). In the promoter region of the human Nrf2 gene (NFE2L2), there are many single-nucleotide polymorphisms (SNPs) that influence Nrf2 expression *in vivo*. Specific age-related disorders, such as acute lung damage, reduced forearm vasodilator response, and Parkinson’s disease, have been linked to these SNPs. Individuals with a variant allele may be more susceptible to the negative effects of medications, have a higher number of comorbidities, and be frailer in the setting of an age-related reduction in Nrf2 (Marzec et al., 2007; Marczak et al., 2012; von Otter et al., 2014). According to a recent study, polymorphism rs35652124 (T>A,C,G) in NFE2L2/Nrf2 gene was found to be associated with aging. Because the G allele is linked to lower NFE2L2/Nrf2 expression, another possible reason for the AA genotype’s higher risk of multimorbidity and frailty is that high NFE2L2 levels are harmful in some disorders. When compared to control mice, Nrf2 knockout animals had a smaller atherosclerotic plaque (Sussan et al., 2008). Furthermore, the rs35652124 AA genotype is linked to a higher risk of high blood pressure and cardiovascular death in adults (Shimoyama et al., 2014). As a result, it is possible that cardiovascular pathology is to blame for the increased levels of multimorbidity and frailty (Scutt et al., 2020).

SIRT2 is one of seven mammalian sirtuins (Sir2-like proteins) that play critical roles in cellular activities such as metabolism and differentiation (Michan and Sinclair, 2007). It is mostly found in the cytoplasm, where it deacetylates -tubulin, but it also migrates to the nucleus during the G2/M phase, where it deacetylates histones, influencing cell cycle progression (North et al., 2003; Vaquero et al., 2006; Inoue et al., 2007). SIRT2 also deacetylates numerous additional substrates (PEPCK1, FOXO1, FOXO3a, p65, and p53) that are involved in key cellular processes linked to organism health, such as homeostasis, oxidative stress management, inflammation, and cell growth and death regulation (de Oliveira et al., 2012). SIRT2 variation rs45592833 (G>T) is located inside a binding region identified by three distinct miRNAs (miR-3170, miR-92a-1-5p, and miR-615-5p), all of which were expected to bind more firmly to the T allele, causing SIRT2 production to be reduced (Crocco et al., 2015). SIRT2 levels have been discovered to be low in various human malignancies, and SIRT2-deficient animals have been reported to develop tumors as they age (Hiratsuka et al., 2003; Kim et al., 2011; Li et al., 2013b). miR-615-5p, which has the highest binding energy change caused by rs45592833, has been found to be deregulated in cancer cell lines, patients with aging-related conditions such as Huntington’s and cardiovascular diseases, and in the muscles of old mice, implying that miR-615-5p downstream targets may be involved in signaling pathways that are important in the aging process (Hulf et al., 2011; Sondermeijer et al., 2011; Hoss et al., 2014; Gao et al., 2015b).

An SNP in the DRD2 gene, rs6276 (A>G), which encodes a G protein-coupled receptor found on postsynaptic dopaminergic neurons, was also found to have a substantial connection with the longevity phenotype. DRD2 signaling is required for the appropriate control of a variety of physiological activities, including locomotion, behavior, and hormone synthesis (Usiello et al., 2000). Six distinct miRNAs were projected to bind to the region containing the polymorphism rs6276 using in silico analysis, with miR-485-5p having the greatest energy binding level to the 3′UTR with the minor G allele (Crocco et al., 2015). As a result, G allele binding is likely to be linked to enhanced miRNA–mRNA binding, resulting in more severe DRD2 expression regulation. DRD2 expression has been found to be downregulated in both striatal and extrastriatal areas of the brain in elderly adults, and that changes in DRD2 receptor density or activity have been linked to age-related declines in motor and cognitive abilities (Noble, 2003; Bäckman et al., 2006; Crocco et al., 2015).

## Conclusion

Dysregulation of miRNAs and their targets is often reported to be involved in cancer progression. Multiple mechanisms for regulation of target gene expression by miRNAs have been proposed. However, recent evidence suggested another layer of complexity in terms of SNPs in either the miRNA seed or their target sequences. These mutations may cause dysregulated gene expression leading to cancer progression. Similar evidence is emerging in diabetes mellitus as well. There is limited scientific literature reporting SNPs in miRNA seed sequences highlighting scope of further exploration.

Overall, the current evidence suggests the need for the in-depth sequence analysis of miRNAs and target genes as well as to correlate the genetic evidence with functional studies. Since single miRNA can target multiple genes and similarly single genes can be targeted by multiple miRNAs, understanding the functional implications of these SNPs can provide new information regarding mechanisms of disease progression.
